# Community Perception towards Mental Illness among Residents of Gimbi Town, Western Ethiopia

**DOI:** 10.1155/2016/6740346

**Published:** 2016-10-20

**Authors:** Misael Benti, Jemal Ebrahim, Tadesse Awoke, Zegeye Yohannis, Asres Bedaso

**Affiliations:** ^1^Psychiatry Department, Gimbi General Hospital, Oromia, Ethiopia; ^2^School of Nursing and Midwifery, College of Medicine and Health Sciences, Hawassa University, Hawassa, Ethiopia; ^3^Department of Biostatistics and Epidemiology, College of Medicine and Health Sciences, University of Gondar, Gondar, Ethiopia; ^4^Research and Training Directorate, Amanuel Specialized Mental Hospital, Addis Ababa, Ethiopia

## Abstract

*Background.* Despite the increased burden of mental health problem, little is known about knowledge and perception of the public towards mental health problems in Ethiopia.* Methods.* Community based cross-sectional study was conducted among selected 845 Gimbi town residents from May 28 to June 28, 2014.* Results.* Out of the total study participants, 304 (37.3%) were found to have poor perception (a score below mean five semantic differential scales for positive questions and above mean for negative questions) of mental illness. Being above 28 years of age (AOR = 0.48 CI (0.23, 0.78)), private workers (AOR = 0.41 CI (0.19, 0.87)), and lack of mental health information were found to be associated with poor perception of mental illness (AOR = 0.133 CI (0.09, 0.20)). Absence of family history of mental illness was also found to be associated with poor perception of mental illness (AOR = 0.37 CI (0.21, 0.66)).* Conclusions.* Significant proportions of the community in Gimbi town were found to have poor perception of mental illness. Poor perception is common among old aged, less educated, private workers, those unable to access mental health information, and those with no family history of mental illness. Mental health education on possible causes, treatment options, and possible outcome of treatment to the community is required.

## 1. Background

Mental and behavioral problem exist in all countries, in women and men at all stages of life, among the poor and rich and among rural and urban people. As many as 450 million people worldwide are estimated to be suffering at any given time from some kind of mental or brain disorder, including behavioral and substance related disorders [1]. Worldwide it is estimated that life time prevalence ranges from 12.2% to 48.6% and 12-month prevalence between 8.25% and 29.1% [2].

Community's perception of mental health varies across the culture, and there are various myths and beliefs regarding mental health [3]. The conceptualization and perceived cause of mental illness vary from community to community. Accordingly, people with mental health problem get different names in different societies [4–6].

People tend to have strong beliefs about the mental illness, and many of these concepts are based on prevailing local systems of belief. Most of the society's perception and attitude towards mental illness are far from the scientific view and this may negatively affect treatment seeking and adherence [7, 8].

Several studies show that people's belief regarding mental illness is also the main factor which leads to stigmatization and labeling. Stigma against people with mental illness remains a significant barrier to positive outcomes across cultures and nations, related to the threat value of mental symptoms, intolerance for diversity, and inaccurate conceptions of mental disorder [9, 10].

Community's perception is dynamic and tends to change as the awareness and education changes. Education and social media are the major factors which move the perception of the community to the scientific perspectives [11].

Globally, including developed and developing countries, people held different explanation regarding mental illness, especially its causal and treatment option. A report of the behavioral risk factor surveillance system shows that in USA 80% of the adult population in the state surveyed agree that mental illness treatment is effective. The rest either disagree or have no idea about that and only 35%–67% of the population agreed that people are caring and sympathetic to people with mental illness [12].

Mental health, neglected for far too long, is crucial to the overall well-being of individuals, societies, and countries and must be universally regarded in a new light. Unfortunately, in most parts of the world, mental health and mental disorders are not regarded with anything like the same importance as physical health [13].

Poor perception of mental illness in different community contributed to low treatment seeking and stigmatization of people with mental illness. They often go to hospitals after they have tried all options and after the symptom has got worse and this in turn negatively affects the prognoses of treatment [14].

Hence, assessing community's perception is important to have appropriate plan of health promotion and scaling up publics' utilization of mental health services, particularly in multiethnic and multicultural Ethiopia as the community's view of mental illness varies with culture.

Studies done in different areas have shown that poor perception towards the mentally ill is mainly deep rooted with various sociodemographic and other factors [7, 8, 10, 13–18].

In Ethiopia there are few published studies [18] assessing community perception towards people with mental illness and no studies were done in Gimbi town concerning this topic. Therefore, this study has great value on assessing the perception of the community towards mental illness. Also the result of this study is very important to the mental health strategy developed so far and to other stakeholders as describing the community's deep rooted belief and perception regarding mental illness in West Wellega, particularly Gimbi town.

## 2. Method and Materials

### 2.1. Study Design and Period

Community based cross-sectional study design was conducted from May 28 to June 28, 2014, at Gimbi town, West Wellega Zone of Oromia region, Ethiopia. The town is located at a distance of 441 Km from the capital city, Addis Ababa to the west. Gimbi town has got two hospitals and one health center. The town has 4 kebeles and according to the 2007 National Housing and Population Census, the projected population of Gimbi town for the year 2014/15 was about 42,286 and the estimated number of households was 8629 [19].

### 2.2. Sampling Techniques

Single population proportion with the assumptions of 95% level of confidence, 5% error, and 50% proportion was used to estimate the minimum sample size for the study. Considering the design effect of two (multiplied by 2) and 10% nonresponse rate, the final sample size became 845.

Following purposive selection of the administrative town (Gimbi town), the two kebeles out of four (the smallest administrative level) were selected randomly. Households in the selected kebeles were approached by systematic sampling method with the first household selected randomly. From each selected household, one individual, 18 years of age and older (based on information from household head), was included for the interview. A lottery method was used to select one individual in a house hold where more than one individual, 18 years of age and older, was found.

### 2.3. Data Collection and Measurements

Data were collected through face to face interview by trained data collectors. A case vignette based standardized questionnaire which explains cases like schizophrenia, major depressive disorder, epilepsy, and generalized anxiety disorder [18] as well as questionnaires adopted from previous studies to assess sociodemographic characteristics and other associated factors was used. Standard 9 items of five semantic deferential scales were also used to assess community perception about mental illness. A score above mean score of five semantic differential scales for positive questions and below mean score for negative questions was considered as having good perception of mental illness. Four trained diploma nurses interviewed the community and were supervised in the field during data collection. Eligible participants not available at home during the first visit of data collection were revisited once on the next day and then registered as nonresponse if not found.

### 2.4. Data Quality Control

The tool was first developed in English language and translated to Amharic language with back translation to English for consistency. Amharic version questionnaire was used to collect data as Amharic is the national official language that majority of Ethiopians speak.

To assure the quality of data the questionnaire was pretested 1 week before the actual data collection time among 40 people not included in the actual sample and appropriate modification was made. The reliability of the tool was found to be *α* = 0.73.

Training on objectives of the study, over all data collection procedure and ethics of the study, was given to data collectors and supervised by principal investigators on the actual data collection site. To ensure confidentiality, interview was conducted in private setting. Throughout the course of the data collection, interviewers were supervised at each site; regular meetings were held between the data collectors and the principal investigator. Two more additional visits were made for eligible respondent not accessed in the first visit. The collected data was reviewed and checked for completeness before data entry.

### 2.5. Data Processing and Analysis

Data clean-up and cross checking were done before analysis. Checked, cleaned, and coded data was entered into EPI info version 7 and exported to SPSS version 20.0 for analysis. Descriptive statistics like frequencies, percentages, mean, and standard deviation were used to present data. Tables and figures were also used to present data. Binary logistic regression was used to see associated factors with the dependent variable. Those variable with *p* value less than 0.02 were entered to multivariate analysis to identify the independent associated factors after confounders are controlled. Finally the variables which had independent association with community perception were identified on the basis of OR, with 95% CI and *p* value less than 0.05.

### 2.6. Ethical Considerations

Ethical clearance was obtained from the Institutional Review Board of University of Gondar and Amanuel mental specialized hospital. Permission letter was obtained from Oromia regional health bureau, West Wellega Zonal health department, and Gimbi town health office hierarchically.

Written informed consent was obtained from each study participant. After reading the consent statement by the data collectors, finger prints were obtained from those participants who could not read and write. The respondents were informed that their inclusion in the study is voluntary and they are free to withdraw from the study if they are not willing to participate.

Also all respondents are informed that they did not have direct payment for their participation in the study. Anonymity was maintained to ensure confidentiality of respondents.

## 3. Results

A total of 845 selected peoples in Gimbi town were interviewed, out of which 29 responses were excluded for gross incompleteness and considered as nonresponse. Therefore, analysis was made based on 816 questionnaires, yielding a response rate of 96.5%.

### 3.1. Sociodemographic Characteristics of Respondents

Among the total respondents, 414 (50.73%) were females and the mean age of participants was 33.0 ± 10 SD. The majority, 706 (86.5%), of study participants were Oromo in ethnicity and four hundred and sixty-seven (57.2%) were Orthodox followers by religion. 577 (70.7%) were married, 285 (34.9%) have elementary level education, and 31.4% of the respondents were merchant. Six hundred and fifteen (75.4%) of the respondents had an average family monthly income less than or equal to 651 Ethiopian birr (Table 1).

### 3.2. Mental Health Information

Mental health information refers to information on the types of mental illness, causes, possibility of treatment, treatment options, and magnitude of mental illness. Accordingly, among all study participants, 564 (69.1%) were reported as having mental health information in the past 12 months while the remaining 252 did not receive any information related to mental health illness. Among 564 respondents having information regarding mental illness 21.3%, 12.4%, and 11% believe that mental illnesses are caused by excessive worry, substance use, and evil spirit, respectively. Mental illness are serious problem (18.5%) and stress can cause mental illness (11.3%), which were among the statements frequently mentioned.

### 3.3. Family History of Mental Illness

**Figure 1:**
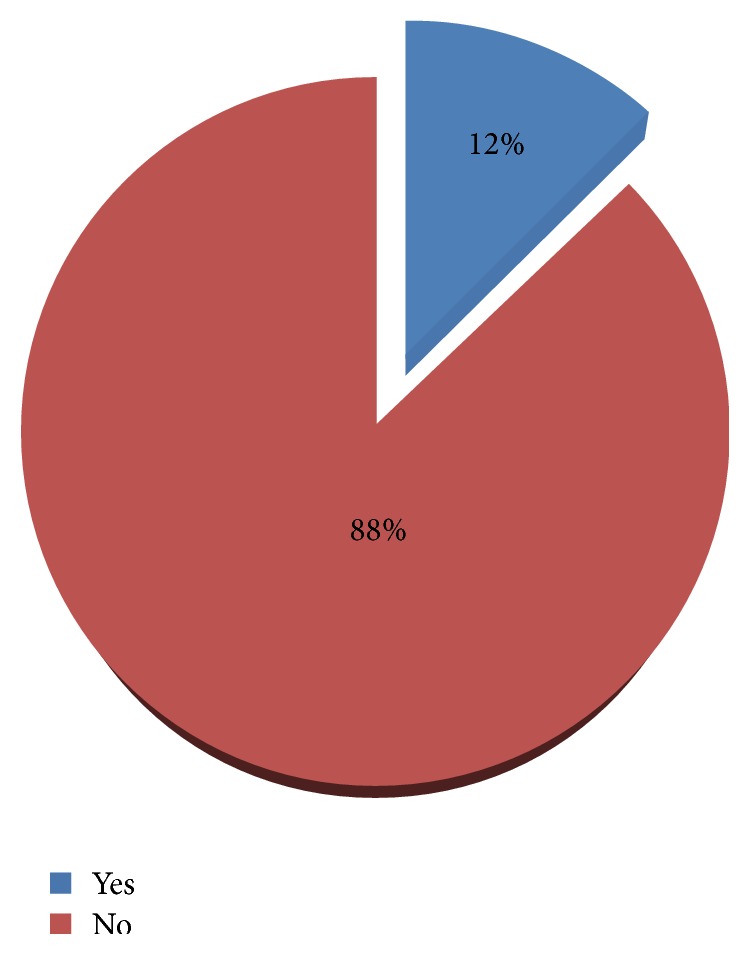
Distribution of family history of mental illness among residents of Gimbi town, 2014.

Presence of family member/s with any type of mental health problem was considered here. Among the total participants, 100 (12.3%) were reported to have family history of mental illness (Figure 1).

### 3.4. Respondents' Perception of Mental Illness

A score below 27 for 9 items of five semantic deferential scales was considered having poor perception towards mental illness in this study. Accordingly (37.3%) respondents were found having poor perception towards mental illness (Table 2).

### 3.5. Respondents' Perceived Cause of Mental Illness

Majority of the respondents attributed the cause of mental illness in the case vignette to supernatural causes like evil spirit, God's punishment, and witchcraft.  Table 3 shows the distribution of perceived cause of mental illness in the case vignettes (Table 3).

### 3.6. Respondents' Recognition of the Case Vignette as Mental Illness

**Figure 2:**
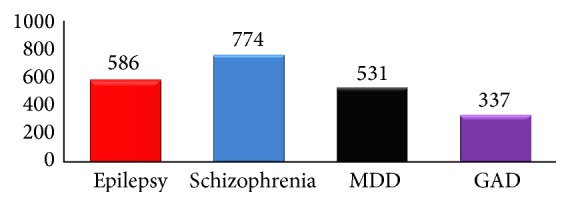
Distribution of respondents' recognition of mental illness among residents of Gimbi town, 2014.

Schizophrenia was the case identified as mental illness by the majority of respondents. Seven hundred and seventy-four (94.9%) of the respondents identified schizophrenia as mental illness. General Anxiety Disorder (GAD) was the least case identified as mental illness by the respondents (Figure 2).

### 3.7. Perception regarding Possibility of Treatment of Mental Illness on the Case Vignette

**Figure 3:**
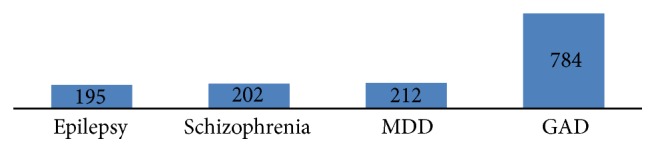
Distribution of respondents' perception regarding possibility of treatment of mental illness (*n* = 816), Gimbi town, 2014.

Great discrepancy was observed among case vignettes regarding the respondents' view of whether or not they can be treated. Majority of the respondents (784) (96.1%), reported that GAD can be treated while 202 (24.8%) of the respondents reported that schizophrenia is treatable (Figure 3).

### 3.8. Respondents Preferred Place of Treatment for the Case Vignettes

Regarding preferred place of treatment: 538 (65.9%), 470 (57.6%), 454 (55.6%), and 254 (31.1%) of respondents prefer health institution for epilepsy, schizophrenia, MDD, and GAD, respectively. See Table 4 for the distribution of the respondents' preferred place of treatment.

### 3.9. Respondents Perceived Severity of Mental Illness

**Figure 4:**
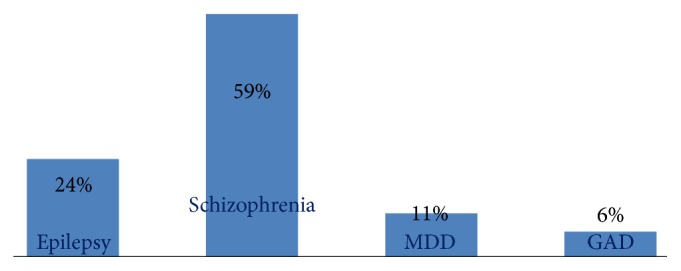
Distribution of respondents' perceived severity of mental illness among residents of Gimbi town, West Wellega Zone, Oromia region, Ethiopia, 2014 (*n* = 816).

Respondents were asked to rank the case vignette from the most serious to the least. Schizophrenia was seen as the most severe by majority of the respondents. Following schizophrenia, epilepsy, major depressive disorder, and generalized anxiety disorder were seen from the most severe to the least, respectively (Figure 4).

### 3.10. Factors Associated with the Respondents' Perception of Mental Illness

In bivariate analyses age of respondents, educational status, occupational status, mental health information, and family history of mental illness were found to be significantly associated with perception of mental illness.

Respondent age, educational level, occupation, mental health information, and family history of mental illness were found to be independently associated after multivariate analysis.

The likelihood of having poor perception of mental illness among those above age 39 is higher than those below 28 years old (AOR = 0.5 (0.30, 0.80)). The proportion of poor perception is observed among those who have no formal education (AOR = 0.09 CI (0.02, 0.46)), elementary education (AOR = 0.07 CI (0.02, 0.33)), and high school level (AOR = 0.13 CI (0.03, 0.61)), when compared with those with degree and above. This shows that perception towards mental illness is higher among educated people.

Community's perceptions of mental illness were also found associated with occupational status. Private workers will have poor perception when compared with government employees (AOR = 0.41 CI (0.09, 0.87)). Mental health information is also associated with perception of mental illness. Having mental health information from any sources is more likely associated with good perception of mental illness (AOR = 0.133 CI (0.09, 0.2)).

The likelihood of having good perception among individual with no family history of mental illness is 63% less when compared with those having family history of mental illness (AOR = 0.37 CI (0.21, 0.66)) (Table 5).

## 4. Discussion

This community based cross-sectional study identified important information on community perception towards mental illness. Significant number of the respondents from Gimbi community had poor perception of mental illness. This study is in line with the study conducted in India where 39.4% of the respondents were found to have poor perception about mental illness [14]. But it is not consistent with study done in Iraq where 30% of the respondents were with poor perception of mental illness [13]. The variation might be due to socioeconomic and cultural difference among respondents.

This study demonstrated that there was higher proportion of poor perception of mental illness among those above 39 years of age compared to the youth. The finding is not consistent with a study done in North Western Ethiopia, Agaro town, where younger respondents were more likely to hold socioenvironmental deprivation responsible for mental and physical illnesses than older respondents [18]. This could be due to the difference in educational level between these two groups or may be due to the fact that the younger respondents may have access to information. Also in study done in Iraq and India no significant association was found with age regarding the community's view of mental illness. This could be due to the tool used or sample characteristics as rural and urban community are involved in both studies [13, 14].

Educational level was found to be one of the sociodemographic characteristics significantly affecting perception of mental illness in this study. Respondents who have no formal education are by 90% more likely to have poor perception when compared with degree holders and above. This finding is in agreement with the study done in Agaro town [18]. Study done in Nigeria also found out that perception of mental illness correlates with educational level [20]. Less educated respondents were more likely to attribute mental illnesses to supernatural retribution. This could be due to poor understanding of scientific explanation regarding causation of mental illness.

Occupational status was significantly associated with perception of mental illness. Government employees will have good perception of mental illness compared to private workers, which implies those in government organization may have more access to current policies, directions, and strategies relatively than those in private organizations. However this finding is not clearly observed in other studies. This may be due to the difference of tool used.

In addition, respondents having information about mental illness from any source were of good perception of mental illness compared to those having no information. This finding is in line with the study done in Karfi region of Nigeria [15]. Theory of psychology also supports this fact. According to psychologists' point of view, perception can be deductive or inductive. If perception is deductive, no probabilistic association needs to be added and no cognition is required. Information is sufficient and deductive perception is possible. If however perception is inductive some premises come from cognition and memory [21].

Family history of mental illness is also another factor found associated with perception of mental illness among Gimbi community. Those who do not have family history of mental illness are found more likely to have poor perception of mental illness than those having family history of mental illness. This may be due to the fact that those having family history of mental illness may share experience. This experience may help them to develop good perception. This factor is also supported by psychologists' explanation. According to Bill, perceptual experiences are experiences of mind-independent things and are themselves an account of the way in which they provide peculiarly basic reasons for beliefs about the world around the perceiver [22].

### 4.1. Conclusion and Recommendation

Significant proportions of community in Gimbi town have poor perception of mental illness. Poor perception of mental illness was common among old age people, those with low educational level, private workers, those who do not have mental health information, and those who do not have family history of mental illness. High level of educational status and access to mental health information strengthen the community's perception of mental illness.

It is important to address the poor perception towards the mentally ill in order to make community based mental health care a reality. The community health professionals should work on awareness creation on mental ill issues at the community level providing relevant information. There is the need to provide more knowledge and increased awareness on the causes of mental illness and also dispel the myths around the causes of the disorders and treatment options.

## Figures and Tables

**Table 1:** Sociodemographic characteristics of respondents (*n* = 816) in Gimbi town, West Wellega Zone, Oromia region, Ethiopia, 2014.

Variables	Category	Frequency	Percentage
Age	18–28	313	38.4
	29–38	301	36.9
	≥39	202	24.8

Sex	Male	402	49.3
	Female	414	50.7

Religion	Orthodox	467	57.2
	Protestant	266	32.6
	Muslim	83	10.2

Ethnicity	Oromo	704	86.3
	Amhara	84	10.1
	Gurage	18	2.2
	Tigre	8	1.2
	Others	2	0.2

Educational status	Degree and above	38	4.7
	Diploma	119	14.6
	High school	280	34.3
	Elementary	285	34.9
	Uneducated	94	11.5

Marital status	Single	221	27.1
	Married	577	70.7
	Divorced	18	2.2

Occupational status	Government	113	13.8
	Private	270	33.1
	Merchant	256	31.4
	Student	66	8.1
	Housewife	111	13.6

Family monthly income in Ethiopian birr	≤450	27	25.4
	451–650	215	26.3
	651–1300	193	23.7
	≥1301	201	24.6

**Table 2:** Distribution of community's perception of mental illness among the residents of Gimbi town, Oromia region, Ethiopia (*n* = 816), 2014.

S. number	Questions	1		2		3		4		5	
		*N*	%	*N*	%	*N*	%	*N*	%	*N*	%
1	Do you agree that substance misuse like alcohol or drug could result in mental illness?	12	1.5	50	6.5	106	13.0	444	55.4	204	25.0
2	Do you agree that genetic inheritance could be the cause of mental illness?	220	27.0	130	15.9	150	18.4	222	27.2	94	11.5
3	Do you agree that head injury can be the cause of mental illness?	52	6.4	130	15.9	111	13.6	308	37.7	215	26.3
4	Do you agree that physical illness (like diabetes, HIV/AIDS) can be the cause of mental illness	54	6.6	221	27.1	49	6.0	273	33.5	219	26.8
5	Do you agree that mental illness is treatable?	12	1.5	220	27.0	60	7.4	304	37.3	220	27.0
6	Do you agree that stress in daily life can cause mental Illness?	19	2.3	107	24.1	65	8.0	270	33.1	265	32.5
7	Do you agree that mental illness is contagious?	435	53.3	152	18.6	171	21	40	4.9	18	2.2
8	Do you agree that mental illness is punishment from God?	38	4.7	88	10.8	400	49.0	226	27.7	64	7.8
9	Do you agree that evil sprite can be the cause of mental illness?	32	3.9	35	4.3	221	27.0	413	50.6	115	14.0

1: strongly disagree; 2: disagree; 3: not sure; 4: agree; 5: strongly agree.

**Table 3:** Distribution of respondents' perceived cause of mental illness among residents of Gimbi town, West Wellega Zone, Oromia region, Ethiopia (multiple responses), 2014.

Causes		Mental illness							
		Epilepsy		Schizophrenia		MDD		GAD	
		*N*	%	*N*	%	*N*	%	*N*	%
Evil spirit	No	262	32.1	232	28.4	280	34.3	334	40.9
	Yes	554	67.9	584	71.6	536	65.7	482	59.1
God's punishment	No	644	78.9	646	79.2	706	86.5	732	89.7
	Yes	172	21.1	170	20.8	110	13.5	84	10.3
Witchcraft	No	648	79.4	640	78.4	668	81.9	684	83.8
	Yes	168	20.6	176	21.6	148	18.1	132	16.2
Daily stress	No	596	73.0	254	31.1	774	94.9	202	24.8
	Yes	220	27.0	562	68.9	42	5.1	614	75.2
Biological	No	736	90.2	710	87.0	204	25.0	185	22.7
	Yes	80	9.8	106	13.0	612	75.0	631	77.3
Substance use	No	630	77.2	394	48.3	632	77.5	696	85.3
	Yes	186	22.8	422	51.7	184	22.5	120	14.7
Physical illness	No	488	59.8	664	81.4	632	77.5	756	92.6
	Yes	328	40.2	152	18.6	184	22.5	60	7.4

**Table 4:** Distribution of respondents preferred place of treatment mental illness among residents of Gimbi town, West Wellega Zone, Ethiopia, 2014.

Treatment option		Mental illness							
		Epilepsy		Schizophrenia		MDD		GAD	
		*N*	%	*N*	%	*N*	%	*N*	%
Family	No	808	99.0	810	99.3	784	96.1	612	75.0
	Yes	8	1.0	6	.7	32	3.9	204	25.0
Hospital/HC	No	278	34.1	346	42.4	362	44.4	562	68.9
	Yes	538	65.9	470	57.6	454	55.6	254	31.1
Holly water	No	658	80.6	656	80.4	658	80.6	728	89.2
	Yes	158	19.4	160	19.6	158	19.4	88	10.8
Traditional healer	No	772	94.6	770	94.4	758	92.9	796	97.5
	Yes	44	5.4	46	5.6	58	7.1	20	2.5
Prayer	No	572	70.1	538	65.9	380	46.6	334	40.9
	Yes	244	29.9	278	34.1	436	53.4	482	59.1
Witchcraft	No	782	95.8	790	96.8	790	96.8	804	98.5
	Yes	34	4.2	26	3.2	26	3.2	12	1.5

**Table 5:** Distribution of factors associated with community's perception of mental illness among residents of Gimbi town, West Wellega Zone, Oromia region, Ethiopia, 2014.

Variable	Perception		Crud OR 95% CI	Adjusted OR
	Good (%)	Poor (%)		
Age				
18–28	215 (68.7)	98 (31.3)	1.00	1.00
29–38	210 (69.8)	91 (30.2)	1.05 (0.7, 1.5)	1.2 (0.8, 1.9)
≥39	87 (43.1)	115 (56.9)	0.35 (0.24, 0.50)	**0.5** (**0.30, 0.80**)^*∗*^
Education				
Illiterate	40 (42.6)	54 (57.4)	0.41 (0.01, 0.18)	**0.09** (**0.02, 0.46**)^*∗*^
Elementary	138 (48.4)	147 (51.6)	0.05 (0.01, 0.22)	**0.07** (**0.02, 0.33**)^*∗*^
High school	187 (66.8)	93 (33.2)	0.11 (0.03, 0.47)	**0.13** (**0.03, 0.61**)^*∗*^
Diploma	118 (93.3)	8 (6.7)	0.77 (0.16, 3.80)	1.35 (0.25, 7.24)
Degree & above	36 (94.7)	2 (5.3)	1.00	1.00
Occupation				
Government	100 (88.5)	13 (11.5)	1.00	RC
Private	121 (44.8)	149 (55.2)	0.11 (0.06, 0.20)	**0.41** (**0.19, 0.87**)^*∗*^
Merchant	188 (73.4)	68 (26.6)	0.36 (0.19, 0.68)	1.25 (0.60, 2.69)
Student	42 (63.6)	24 (36.4)	0.23 (0.11, 0.49)	0.62 (0.25, 1.56)
Housewife	61 (55.0)	50 (45.0)	0.16 (0.08, 0.32)	0.84 (.36, 1.96)
Mental health information	Yes 423 (75.0)	141 (25.0)	1.00	RC
	No 89 (35.3)	161 (64.7)	0.18 (0.13, 0.25)	**0.133** (**0.09, 0.2**)^*∗*^
Family history of mental illness	Yes 74 (74.0)	26 (26.0)	1.00	RC
	No 438 (61.2)	278 (38.8)	0.14 (0.35, 0.89)	**0.37** (**0.21, 0.66**)^*∗*^

^*∗*^ *p* value < 0.05; RC: reference category.
